# Method for Determining the Plasmon Resonance Wavelength in Fiber Sensors Based on Tilted Fiber Bragg Gratings

**DOI:** 10.3390/s19194245

**Published:** 2019-09-30

**Authors:** Egor Manuylovich, Kirill Tomyshev, Oleg V. Butov

**Affiliations:** Kotelnikov Institute of Radioengineering and Electronics of RAS, 125009 Moscow, Mokhovaya 11-7, Russia; manuylovich@phystech.edu (E.M.); obutov@mail.ru (O.V.B.)

**Keywords:** tilted fiber Bragg gratings, optical fiber sensors, plasmon sensors, data processing

## Abstract

Surface plasmon resonance-based fiber-optic sensors are of increasing interest in modern sensory research, especially for chemical and biomedical applications. Special attention deserves to be given to sensors based on tilted fiber Bragg gratings, due to their unique spectral properties and potentially high sensitivity and resolution. However, the principal task is to determine the plasmon resonance wavelength based on the spectral characteristics of the sensor and, most importantly, to measure changes in environmental parameters with high resolution, while the existing indirect methods are only useable in a narrow spectral range. In this paper, we present a new approach to solving this problem, based on the original method of determining the plasmon resonance spectral position in the automatic mode by precisely calculating the constriction location on the transmission spectrum of the sensor. We also present an experimental comparison of various data processing methods in both a narrow and a wide range of the refractive indexes. Application of our method resulted in achieving a resolution of up to 3 × 10^−6^ in terms of the refractive index.

## 1. Introduction

Surface plasmon resonance (SPR) is a subject of modern scientific research [1,2,3,4,5]. Various sensors and measuring complexes based on this phenomenon are used in physical, chemical, and biological research and analysis, including immunoassay systems [1,2,3]. The general principle of such systems’ operation is based on the physical nature of the phenomenon itself. The key point here is the high degree of dependence of the surface plasmons’ dispersion ratio on the refractive index of the external medium. Any changes in this index near the metal surface, whether changes in the composition of the environment or surface modification of the sensor itself due to its interaction with the environment, will immediately be reflected in the resonance wavelength.

Surface plasmon excitation can be performed by means of optical radiation incident at an angle θ to the metal surface. If the equality condition for the projection of the incident radiation’s wave vector and the wave vector of the surface plasmon propagating over the metal surface is satisfied at a given wavelength, the effect of the SPR is observed, and the energy of the exciting light transfers effectively into the plasmon’s energy.

This principle underlies the operation of the classical Kretschmann [6] and Otto [7] schemes. These schemes utilize the correlation between the reflected laser beam intensity and the angle of incidence to determine plasmon resonance. Plasmon resonance is considered to occur at the minimum of reflection intensity. This effect is observed under the condition of phase synchronism, i.e., the coincidence of the plasmon wave vector and the projection of the optical radiation’s wave vector to the surface at a given frequency. By changing the incident angle, one can track changes in the nearest environment of the plasmon sensor with high accuracy. Based on these schemes, immunoassay complexes for the high-precision determination of low concentrations of protein molecules in organic solutions have already been produced [1,4,5,8,9]. The sensor’s surface modified by antibodies is extremely sensitive to the corresponding antigens. During the antigen–antibody interaction, the physical properties of the nearest environment of the plasmon sensor change, resulting in a change in its indications.

**Figure 1:**
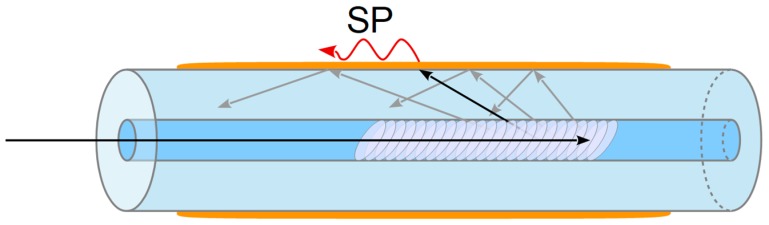
Scheme of an SPR fiber-based TFBG-assisted sensor.

Plasmon resonance in optical fibers is of particular interest. Using such advantages of fiber-optic sensors as mobility, compactness, and convenience of application in microfluidic systems, it is possible to create promising highly sensitive fiber-based plasmon sensors, including those for biomedical applications [3,10,11,12,13,14,15,16,17,18,19,20,21,22,23]. To satisfy the plasmon resonance excitation conditions in optical fibers, it is necessary to guide the optical radiation energy to the outer surface of the fiber, which is the working surface of the sensor. For this, a number of methods can be used, such as thermal taping, polishing or chemical etching of the fiber [10]. However, methods based on the use of tilted fiber Bragg gratings (TFBG) are particularly interesting [24,25,26,27,28,29,30,31,32,33,34,35,36,37,38,39,40,41,42,43,44,45]. A schematic illustration of such a structure is shown in Figure 1.

**Figure 2:**
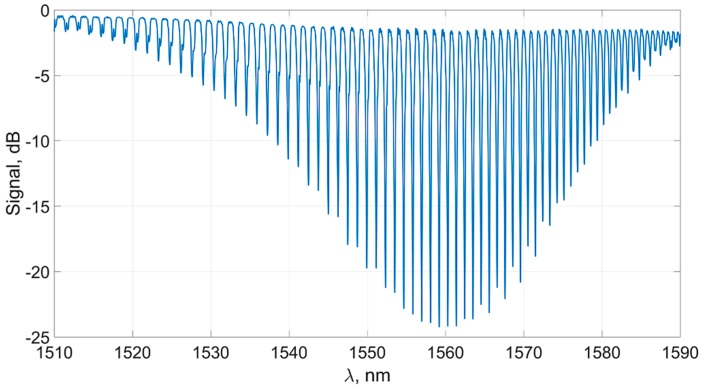
TFBG transmission spectrum.

Such a grating effectively excites a discrete set of cladding modes [36]. The propagation velocities of these modes have different projections onto the fiber surface. A typical transmission spectrum of such a structure is shown in Figure 2.

**Figure 3:**
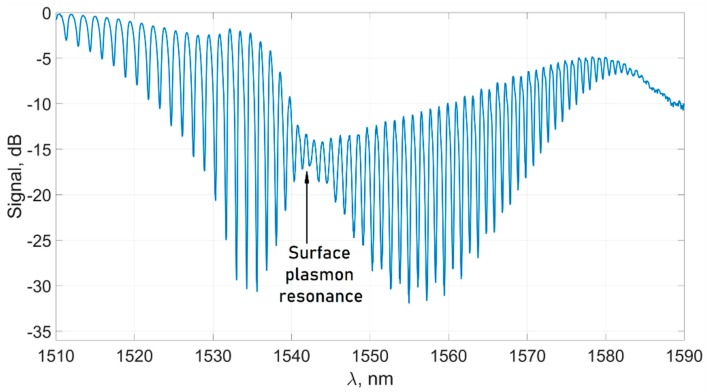
Typical transmission spectrum of the plasmon TFBG-assisted sensor.

If the cylindrical surface of the fiber is covered with a layer of gold about 40 nm thick, the required conditions for the SPR are created. As in the case of the classical Krechmann scheme, the resonance condition is satisfied for certain cladding modes. In this case, energy is efficiently transferred from the cladding modes to the surface plasmon. This process is reflected in the transmission spectrum of the tilted grating in the form of a characteristic “constriction”, which is a narrowing of the pattern of spectral peaks and dips (Figure 3). If the refractive index of the environment changes, the magnitude of the plasmon wave vector also changes; therefore, the spectral position of the “constriction” changes [10]. Obviously, parameters such as resolution and limit of detection of the sensor depend both on the stability of the sensor itself and on the accuracy of determining the plasmon resonance wavelength from the experimentally measured transmission spectrum. If the first task can be solved by mechanical stabilization of the sensor, then to solve the second one, it is necessary to use the original mathematical apparatus, which will be able to clearly identify and interpret small changes in the transmission spectrum of the sensor.

Despite the large number of publications devoted to this kind of sensor, a universal way of tracking changes in the plasmon resonance wavelength with high accuracy has not yet been found. There are methods for determining changes in the concentration of the measured substance by measuring the intensity of individual spectral peaks near the resonance wavelength. Thus, in [22,28,29,30,31,32,33,46], analyzing the intensity or spectral position of one or two peaks located near the spectral “constriction” was proposed. Indeed, in the case of observing small changes in the refractive index of the environment, such methods can give a relatively high resolution and detection limit. On the other hand, the use of such methods for large changes in the refractive index can lead to significant errors because it becomes necessary to switch to other spectral peaks as the plasmon resonance wavelength shifts. As a result, the sensor’s readings become unstable. This method is not universal and requires individual calibration for each sensor. Moreover, methods for determining the intensity of individual spectral peaks can be sensitive to the spectral noise of both the signal source and the analyzer and, as a result, show limited accuracy and reproducibility of readings. As it is known, the actual resolution of such methods in terms of the refractive index does not exceed 10^-5^, including a small dynamic range of the sensor [31,32,33].

In this paper, we present the description of several new, universal methods for determining the wavelength of the plasmon resonance, united by a common mathematical idea based on analyzing the large array of spectral points. We also compare the accuracy of determining the refractive index with our methods and the “traditional” method based on measuring the height of an individual spectral peak.

## 2. Experiments

In our work, we used sensors based on tilted Bragg gratings with a slope of about 11°. Bragg gratings were inscribed in a standard Corning SMF-28e telecommunication optical fiber, 125 μm in diameter. The length of the grating was about 10 mm. The fiber section with the inscribed Bragg grating was covered with a gold layer, 40 nm thick, with preliminary deposition of a chromium underlayer 2–3 nm thick. The coating was carried out by the method of thermal evaporation of metal under vacuum conditions, followed by deposition on the fiber’s cylindrical surface. The uniformity of the coating was achieved by the rotation of the fiber around its axis during the deposition process with a period of full rotation many times lower than the evaporation time of the metal [24,25,26].

**Figure 4:**
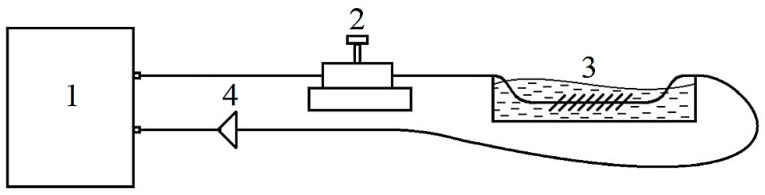
Experimental setup. **1**—multichannel interrogator, **2**—mechanical polarization controller, **3**—tank with the sensor immersed in liquid, **4**—optical fiber isolator.

The experimental setup is shown in Figure 4.

The sensors’ transmission spectra were obtained by means of a MicronOptics SM-125 Bragg interrogator with a scan range of 1510–1590 nm. To obtain the transmission spectra, two channels of the interrogator were used, one of which featured an isolator blocking the reflected signal. As only one polarization of light effectively interacts with the gold surface [6,10,26], a polarization controller was installed before the sensor element to maintain the polarization state. The sensor itself was immersed in a tank with the test solution.

## 3. Method Description

### 3.1. The Idea of the Processing

The idea behind the new processing methods is to determine the superposition of the set of peaks in the spectrum of the sensor near the plasmon resonance wavelength by approximating their intensities with an analytical curve. From the parameters of this curve, the plasmon resonance wavelength is calculated. The spectral position and amplitude of the peaks close to the resonance wavelength depend strictly on the refractive index of the external medium. Because a number of spectral peaks are involved in determining this wavelength, the measurement error is significantly reduced.

**Figure 5:**
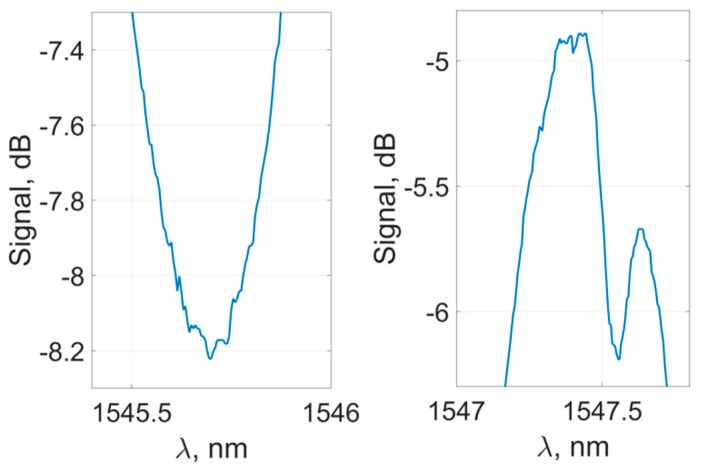
Shape of the sensor’s spectral peaks and features preventing the accurate determination of their characteristics.

In their original form, the experimental transmission spectra of the sensor are inapplicable for the peaks’ characteristics determination, as each individual peak is highly subjected to noise and specific spectral bifurcation due to the interaction features of cladding and core fiber modes [45] (Figure 5). Such features may induce significant distortions during the determination of the peaks’ spectral position. To mitigate the effect of these perturbations, each of the peaks can be approximated by an analytical curve with a characteristic spectral maximum (such as Gaussian or parabola), or the signal can be filtered. Experiments with various methods showed the preferred usage of the filtering method, which we applied in our work. The signal features were mitigated during the preprocessing described in detail below.

### 3.2. Signal Preprocessing

At the first stage of spectrum processing, Fourier filtering of the spectra is done. During this process, the perturbations of the peak shape are smoothed out, and the spectrum is aligned with the horizontal line.

Similar to the filtering of time signals, we will call Fourier space a generalized frequency space [47]. Note that these generalized frequencies are not related to the frequency of the optical radiation and refer to the characteristic period of the intensity change in the spectrum.

(1) $$S_{f}=\frac{1}{2\pi }\int_{\zeta }^{\xi }\left[\int_{0}^{+\infty }S\exp(-i\lambda \varpi )d\lambda \right]\exp(i\lambda \varpi )d\varpi$$

**Figure 6:**
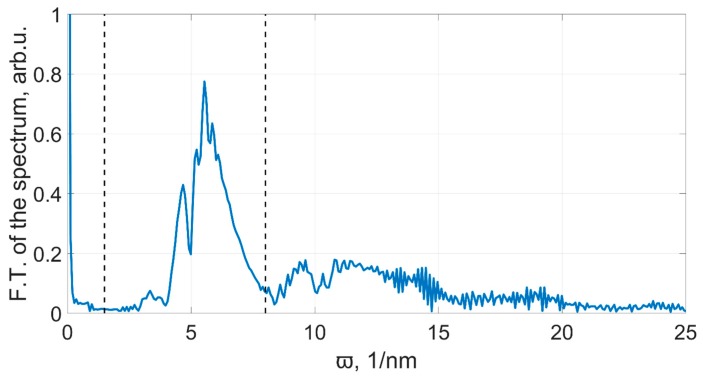
The Fourier transform of the sensor’s spectrum, presented in the generalized frequency space.

Here, *S* is the spectrum of the sensor signal, *λ* is the wavelength in nanometers, ϖ is the generalized frequency, *ζ* is the lower generalized filtering frequency, and *ξ* is the upper generalized frequency. The Fourier transform of the spectrum is presented in Figure 6. While processing, we keep only the part that lies between the vertical dashed lines.

In the software implementation, the fast Fourier transform is used, and filtering is performed by restricting the series of samples in the generalized frequency space, which corresponds to the rectangular mask short-time Fourier transform, which means nullifying the components of the corresponding generalized frequencies.

Fourier filtering with low generalized frequencies suppression aligns the spectrum with the horizontal line. The lower limit of the generalized frequency is determined from the linearity condition of the filtered out signal in the region of the plasmon resonance wavelength.

(2) $$\underline{S}=\frac{1}{2\pi }\int_{0}^{\zeta }\left[\int_{0}^{+\infty }S\exp(-i\lambda \varpi )d\lambda \right]\exp(i\lambda \varpi )d\varpi$$

**Figure 7:**
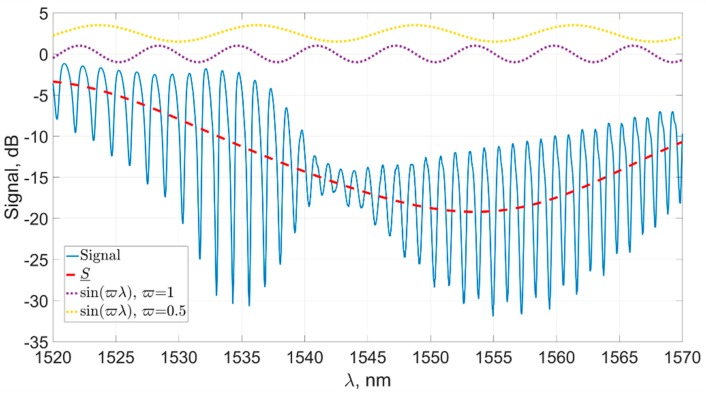
The original signal (solid) and its component *S*, filtered in the process of signal alignment with the horizontal axis (dashed), and components with generalized frequencies ϖ=0.5 and ϖ=1 (dotted).

Figure 7 shows the source signal and its filtered component *S* at *ζ* = 0.5, which, as was shown by our experiments, is the optimal value for the spectra of the sensors we used. In addition, the figure shows the components corresponding to the generalized frequencies ϖ=0.5 and ϖ=1 for a visual representation of the parameter used.

The choice of the optimal lower filtering frequency may depend on the properties of the sensor used, in particular, on the tilt angle of the Bragg grating. When choosing a lower filtering frequency, the smoothness of the filtered component in the region of the plasmon resonance wavelength should be taken into account. Choosing an overestimated value for this parameter can result in a change in the relative intensity of the peaks located near the plasmon resonance, which has a negative effect on the accuracy and linearity of the plasmon resonance wavelength shift determination.

**Figure 8:**
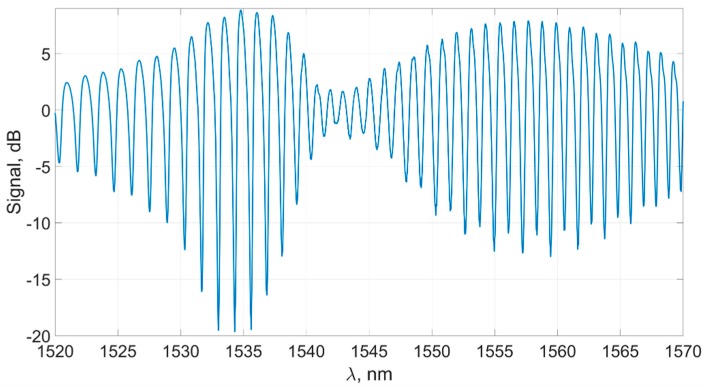
Sensor signal after alignment with the horizontal axis.

The result of the lower frequency filtering is shown in Figure 8.

Note that after aligning the signal using low-pass filtering, perturbations of spectral peaks are still present. To smooth them, Fourier filtering is performed while cutting off high generalized frequencies.

(3) $$S_{f}=\frac{1}{2\pi }\int_{\xi }^{+\infty }\;\left[\int_{0}^{+\infty }\underline{S}\exp(-i\lambda \varpi )d\lambda \right]\exp(i\lambda \varpi )d\varpi$$

**Figure 9:**
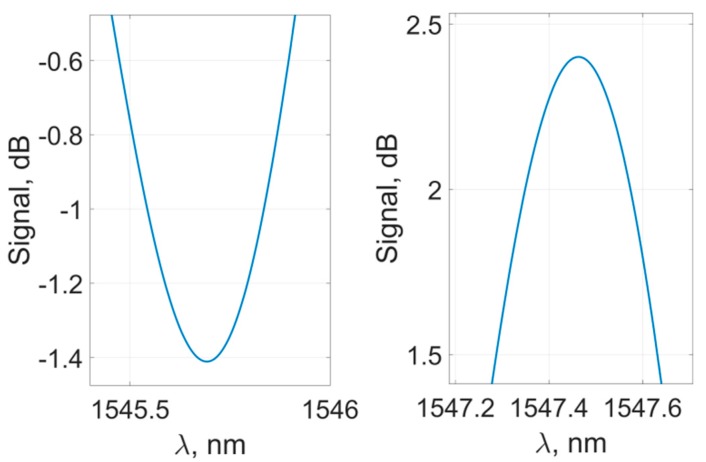
Signal peaks after filtering.

**Figure 10:**
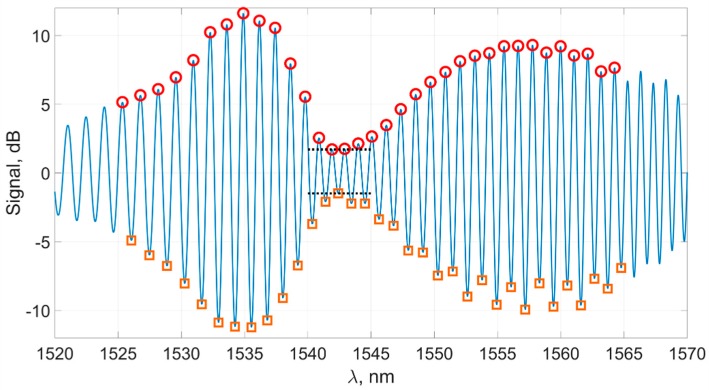
The filtered spectrum of the sensor’s signal with detected peaks lying in the region of the plasmon resonance wavelength: the upper group $Ext^{top}$ (circles), the lower group $Ext^{bot}$ (squares), and the amplitude of the constriction (dotted).

For the sensors used, it was established experimentally that the optimal value of the upper generalized filtering frequency lies in the range of *ξ* = 6–9, depending on the spectral characteristics of the sensor and the interrogator. With such a value of *ξ*, all the noises and perturbations of the multimode interaction are smoothed, but all the characteristic features of the spectrum inherent in the effect of plasmon resonance remain, as shown in Figure 9 and Figure 10.

### 3.3. Plasmon Resonance Wavelength Calculation

After the initial filtering is completed, the plasmon resonance wavelength is calculated in several stages.

At the first stage, the positions of the spectral peaks are determined. After filtering, the spectrum becomes a smooth curve, and it is convenient to calculate the peak positions as points where the derivative changes sign. The calculation of the derivative is carried out numerically. The most important part of the spectrum is near the plasmon resonance wavelength. We denote this part of the spectrum Λ. In the experiment, the magnitude of the shift usually does not exceed 10 nm in each direction and so, for the sensor whose spectrum is shown in Figure 3, it is more than enough to take the region Λ from 1530 to 1570 nm. It should be noted that for the further algorithm of determining the plasmon resonance wavelength, the search region Λ is set so that the spectral waist gets into it, and in the whole Λ region, the amplitude of the spectral peaks has a minimum in the area of the plasmon resonance wavelength.

Figure 10 shows the spectrum of the sensor with the calculated coordinates of the peaks in the required region. We denote all peaks in the spectrum as *Ext*: the upper group of peaks will be $Ext^{top}$, and the lower group of peaks will be $Ext^{bot}$. Their coordinates, respectively, are $Ext_{x}$ and $Ext_{y}$.

The next step is to preliminarily determine the region of the plasmon resonance wavelength by determining the x coordinates of the peak closest to the spectral constriction. Thus, the preliminary method is set as the abscissa of the peak from the upper group having the smallest value of the ordinate.

(4) $$\lambda_{0}^{SPR}=Ext_{x}^{top}\left(Ext_{y}^{top}=\min(Ext_{y}^{top})\right)$$

The accuracy of this method does not exceed the average distance between the peaks in the spectrum and is approximately equal to 1 nm. It is only used as an initial fitting for other methods. More precise methods are based on approximating the coordinates of the peaks located in the previously chosen region of the spectrum Λ near the plasmon resonance wavelength $\lambda_{0}^{SPR}$. We have considered several methods for such an approximation.

**Figure 11:**
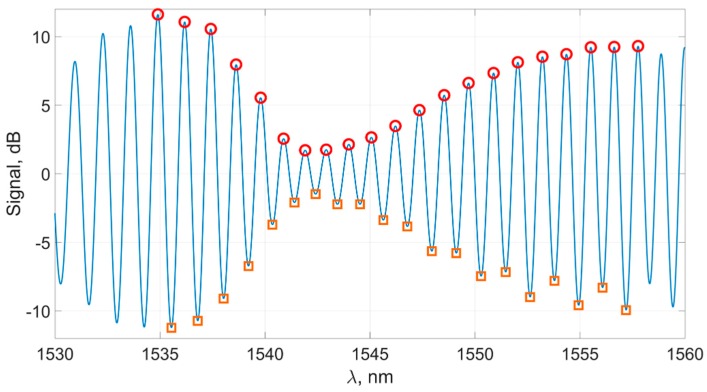
The filtered spectrum of the sensor’s signal and the subgroups $\overset{⌢}{E}xt^{top}$ (circles) and $\overset{⌢}{E}xt^{bot}$ (squares).

For further processing, it is convenient to allocate subgroups $\overset{⌢}{E}xt^{top}$ and $\overset{⌢}{E}xt^{bot}$ from groups $Ext^{top}$ and $Ext^{bot}$, by which we denote the groups of peaks on the “inner slopes” of the spectrum near the constriction. Subgroups $\overset{⌢}{E}xt^{top}$ and $\overset{⌢}{E}xt^{bot}$ are shown in Figure 11.

We will fit these subgroups by smooth curves.

It should be noted that, as a rule, in the experiment, it was important to measure the magnitude of the plasmon resonance wavelength shift, rather than its absolute value. In our experiments, we used a set of spectra that were processed using three similar methods.

The first method was to fit the subgroup of peaks $\overset{⌢}{E}xt^{top}$ by a function of the form

(5) $$f_{\left(1\right)}=h-\beta_{1}\exp\left(-\beta_{2}{\left(x-\beta_{3}\right)}^{2}\right)$$

Here, h is the height of the horizontal asymptote—a parameter defined for all spectra in the series—and $\beta_{i}$ are the parameters determined by the least squares method for each spectrum. The parameter h is also determined by the least squares method while fitting the first spectrum of the series by Function (5). Thus, the curves of (5) are inverted Gaussians and are stated by three independent parameters. The minimum of the analytic Function (5) will be called the plasmon resonance wavelength, determined by the method $f_{\left(1\right)}$:

(6) $$\lambda_{1}^{SPR}=\arg\left(f_{\left(1\right)}=\min(f_{\left(1\right)})\right)=\beta_{3}$$

This method is insensitive to the change in the steepness of the right and left slopes of the spectrum envelope; however, it allows us to track the shift of the spectral constriction as a whole. This method can work well with small changes in the refractive index of the external environment, when the shape of the constriction does not change while the wavelength of the plasmon resonance shifts. The advantage of this method is its relative simplicity due to the small number of variable parameters.

The second alternative method that can be used for calculations is to fit the upper part of the extremes with the analytical curve:

(7) $$f_{\left(2\right)}=\frac{\beta_{1}}{1+\exp\left(\beta_{2}(x-\beta_{3})\right)}+\frac{\beta_{4}}{1+\exp\left(-\beta_{5}(x-\beta_{6})\right)}$$

**Figure 12:**
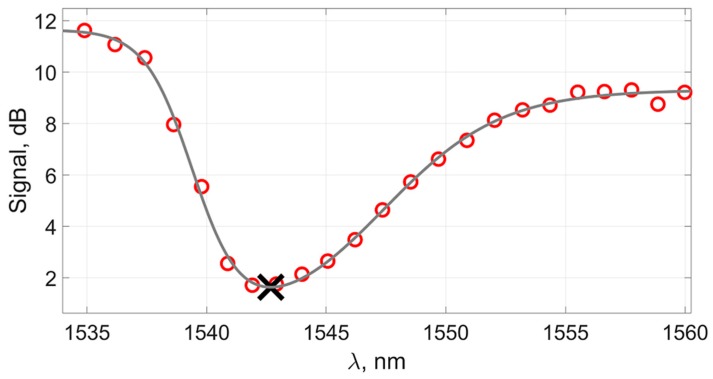
A subgroup $\overset{⌢}{E}xt^{top}$ (circles), smooth function $f_{\left(2\right)}$ fitting this subgroup, and its minimum (cross).

This is a six-parameter curve; here, $\beta_{i}$ are the parameters determined by the method of least squares for each spectrum while approximated by Function (7). Curves of the form (7) take into account the steepness of the graph’s envelope both to the right and to the left of the constriction and fit the group $\overset{⌢}{E}xt^{top}$, which consists of about 20 points (Figure 12).

The plasmon resonance wavelength for this method is calculated as

(8) $$\lambda_{2}^{SPR}=\arg\left(f_{\left(2\right)}=\min(f_{\left(2\right)})\right)$$

This method takes into account the shape (steepness) of the right and left slopes in the spectral dip, which, as expected, will make it possible to carry out measurements more correctly in a wide range of refractive indexes, because it takes into account the change in the shape of the spectrum envelope.

We have introduced one more method that uses both $\overset{⌢}{E}xt^{top}$ and $\overset{⌢}{E}xt^{bot}$ groups as input data and, at the same time, has 12 independent parameters:

(9) $$\begin{array}{l} f_{\left(3\right)}^{top}=\frac{\beta_{11}}{1+\exp\left(\beta_{21}(x-\beta_{31})\right)}+\frac{\beta_{41}}{1+\exp\left(-\beta_{51}(x-\beta_{61})\right)}\\ f_{\left(3\right)}^{bot}=\frac{-\beta_{21}}{1+\exp\left(\beta_{22}(x-\beta_{32})\right)}+\frac{-\beta_{42}}{1+\exp\left(-\beta_{52}(x-\beta_{62})\right)} \end{array}$$

Here, $f_{\left(3\right)}^{top}$ is the curve fitting the subgroup $\overset{⌢}{E}xt^{top}$, and $f_{\left(3\right)}^{bot}$ is the curve fitting the subgroup $\overset{⌢}{E}xt^{bot}$. Parameters $\beta_{ij}$ are determined by the Nelder–Mead method [48], with minimization of the standard deviation of both curves from the corresponding groups. The method uses 12 independent parameters to fit about 40 points with two curves.

The point of intersection of the zero horizontal line and the segment connecting the extremes of the functions $f_{\left(3\right)}^{top}$ and $f_{\left(3\right)}^{bot}$ will be called the plasmon resonance wavelength $\lambda_{3}^{SPR}$, determined by the method $f_{\left(3\right)}$:

(10) $$\begin{array}{l} \lambda_{3}^{top}=\arg\left(f_{\left(3\right)}^{top}=\min(f_{\left(3\right)}^{top})\right)\\ \lambda_{3}^{bot}=\arg\left(f_{\left(3\right)}^{bot}=\max(f_{\left(3\right)}^{bot})\right)\\ \lambda_{3}^{SPR}=f_{\left(3\right)}^{top}\left(\lambda_{3}^{top}\right)\frac{\lambda_{3}^{top}-\lambda_{3}^{bot}}{f_{\left(3\right)}^{bot}\left(\lambda_{3}^{bot}\right)-f_{\left(3\right)}^{top}\left(\lambda_{3}^{top}\right)}+\lambda_{3}^{top} \end{array}$$

**Figure 13:**
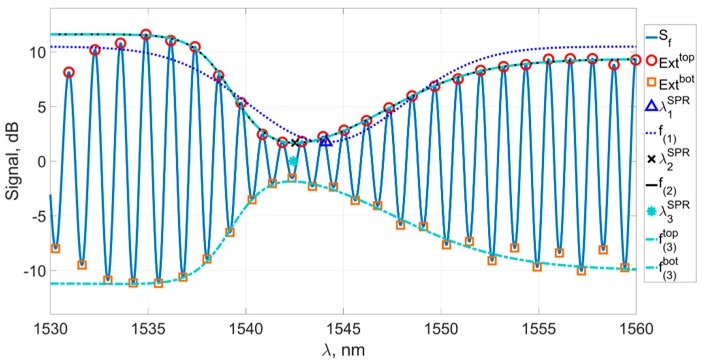
The filtered spectrum of the sensor’s signal, subgroups $Ext^{top}$ (circles) and $Ext^{bot}$ (squares) and their fitting curves, corresponding to different methods.

Figure 13 shows the filtered spectrum of the sensor, the groups of peaks, the functions fitting them, and the plasmon resonance wavelengths determined by the methods described above. It can be seen from the figure that the minima of the fitting curves differ significantly from each other; however, this does not mean that it is impossible to use any of those functions for calculations and does not demonstrate their inefficiency. As noted above, it is not the absolute value of the plasmon resonance wavelength that is important but the precise measurement of its shift with a change in the refractive index of the environment.

The described methods have similar principles for determining the plasmon resonance wavelength. The first method has the minimum number of fitting parameters and rather poorly fits the upper extremum group. It does not allow for the changes to be tracked in the shape of the transmission spectrum of the sensor near the plasmon resonance while making measurements in a large range of refractive indexes. It describes the displacement of the constriction as a whole. However, this method has relative simplicity and, as noted above, can be used to track small changes in the refractive index of the surrounding environment.

The second method has six fitting parameters for approximation of about 20 points. It describes the shape of the constriction’s upper part rather well and takes into account the different steepness of the spectrum envelope’s right and left slopes and the dependence of the envelope form on the plasmon resonance spectral position. This allows the application of the second method with large changes in the refractive index while not losing the resolution of the sensor.

Finally, the third method allows us to track changes in the shape and height of the horizontal asymptotes of both the upper and lower groups of extremes, which also allows this method to work with large changes in the plasmon resonance wavelength. The latter method uses 12 independent fitting parameters to approximate about 40 points in each spectrum. The additional usage of the lower envelope makes the calculations more complicated; on the other hand, it can provide an increase in the accuracy of measurements over a small range due to the processing of the bigger number of significant spectral reference points.

## 4. Results and Discussion

In the experiments, the response of the sensor to changes in the concentration of an aqueous solution of isopropyl alcohol was measured. In the first case, the measurements were carried out in a small concentration range with a small step between the experimental points. Mass concentration varied from 0 to 0.078, which corresponds to a change in the refractive index of the solution in the range of 6.8 × 10^−3^. The initial spectrum of the sensor immersed in water is presented in Figure 3. The refractive indexes of the solutions used in our experiments were calculated from the data presented in paper [49] corresponding to the solution concentration. We assumed that the solution’s refractive index change at the 1.5 µm range for small concentrations is similar to the visible range data presented in [49].

In the series of measurements, each spectrum was processed in accordance with methods 1–3 described above. For comparison, the results of the “classical” method are presented, in which the change in the refractive index of the medium is measured by the change in the height of the selected peak. For the sensor used in the first series of experiments, the peak of the original signal near 1541 nm was chosen because it is located on the slope of the envelope of the graph not far from the constriction, and its height changes quite strongly with the changes of the SPR-wavelength in the actual solution concentration range.

**Figure 14:**
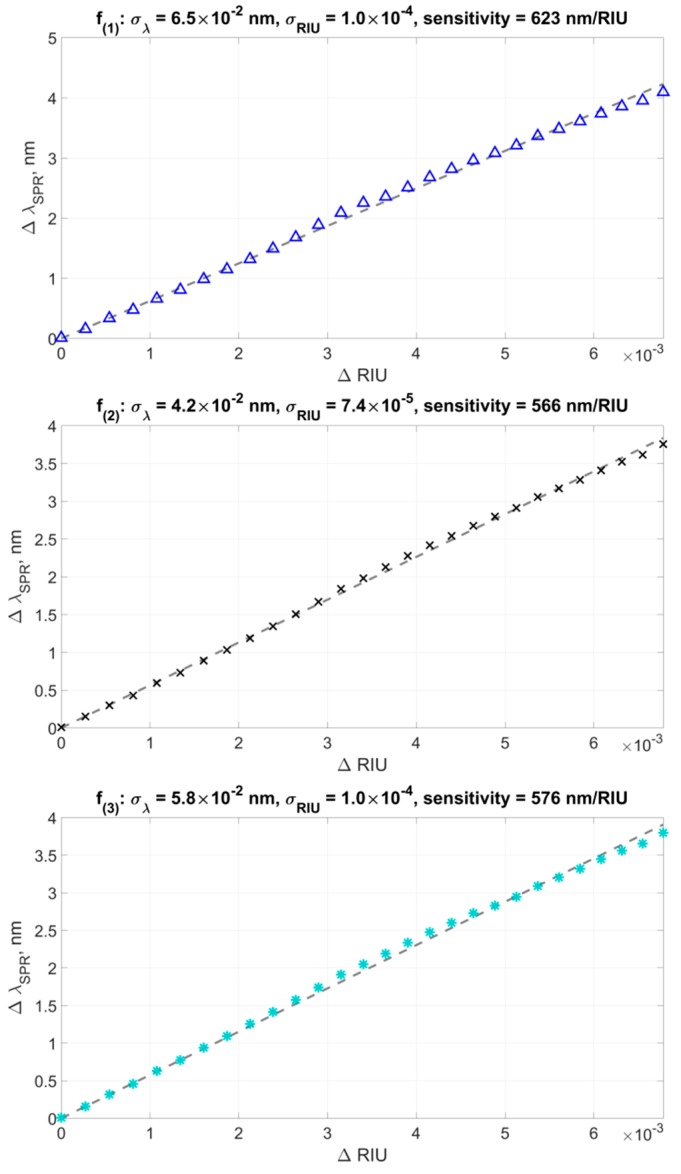
Dependence of the SPR wavelength shift calculated from the data of the first experiment according to methods $f_{\left(1\right)}$ (a), $f_{\left(2\right)}$ (b), $f_{\left(3\right)}$ (c) on the change in the refractive index.

Figure 14 shows the changes in the plasmon resonance wavelength found by three different methods from the ΔRIU values. The experimental data was fitted by a linear function g(ΔRIU) passing through the zero point (0, 0), corresponding to pure water. It should be noted that the deviation of the experimental points from the direct approximation is due to the sequential dilution inaccuracy of the alcohol solution in water, which makes a significant contribution to the value of the standard deviation. However, we can use the total standard deviation to conduct a relative comparison of various data processing methods. The slope of the direct approximation determined the sensor sensitivity value obtained for each data processing method.

Standard deviation was calculated as the standard deviation of experimental points from the curve approximating them:

(11) $$\sigma_{\lambda }=\sqrt{\frac{1}{N}{\sum_{i=1}^{N}\left(\lambda_{SPR}^{\left(i\right)}-g\left(\Delta RIU^{\left(i\right)}\right)\right)}^{2}}$$

For the first method, the standard deviation of points from a straight line was $\sigma_{\lambda }^{\left(1\right)}$= 0.065 nm at the sensor sensitivity of 623 nm/RIU. Therefore, in terms of the refractive index units, the standard deviation was 10^−4^ RIU. However, as noted previously, this method operates well only in a narrow range of refractive indexes because it does not take into account changes in the shape of the envelope.

The second method demonstrated the better value of the standard deviation of points from a straight line, which was $\sigma_{\lambda }^{\left(2\right)}=0.042\;\mathrm{nm}$ at the calculated slope of the curve at 566 nm/RIU, which means a deviation of about 7.4 × 10^−5^ RIU in terms of the refractive index.

The third processing method showed a comparatively worse result for the standard deviation, which amounted to a value of $\sigma_{\lambda }^{\left(3\right)}=0.058\;\mathrm{nm}$, and with a calculated sensitivity of 576 nm/RIU, it showed a deviation value of 10^-4^ RIU in units of the refractive index. Comparatively worse results for the standard deviation appear due to the fact that the lower extrema are less stable, thus introducing a large error in the calculation of the lower six-parameter function when operating in a wide range of refractive indexes.

The sensor sensitivity values calculated by the second and third methods are close to each other and correspond to known literature data [5,10,32,34]. The increased sensitivity in the first method, as noted in Section 3, is explained by the peculiarities of its mathematical apparatus, which does not take into account the shape of the transmission spectrum envelope of TFBG and, accordingly, may give an error when calculating the change in the plasmon resonance wavelength.

**Figure 15:**
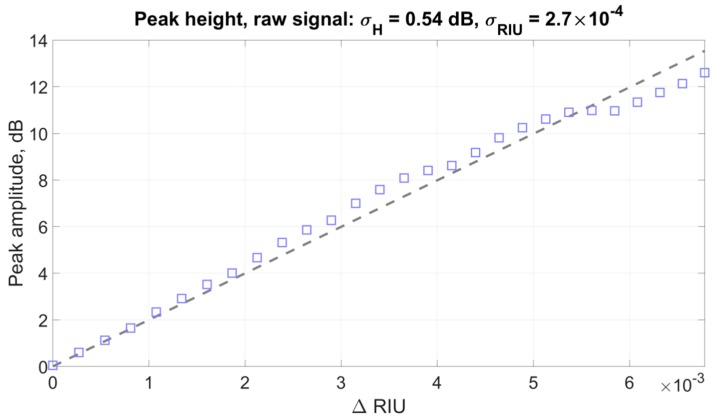
Dependence of the peak height in the region of 1541 nm on the change in the refractive index and the straight approximating line for the “classical” method applied to the original signal.

Figure 15 presents the results of the experimental data processing using the amplitude of the spectral peak near 1541 nm.

The graph shows that there are significant deviations from the linear behavior of the experimental dependence. In this case, the standard deviation of the points from the straight line was *σ_dB_* = 0.54 or 2.7 × 10^−4^ in terms of the refractive index, which is noticeably worse than any of the other presented methods.

Obviously, the method of monitoring the changes in the plasmon resonance wavelength using the amplitude of the nearby peak and its variations can be applicable only in a small measurement range, as with the first method proposed in this paper.

Table 1 shows the standard deviation of the refractive index for the same experimental data for the results processed by different methods. Once again, it should be noted that these values of the standard deviation are not characteristic of the sensory system and do not demonstrate its resolution or detection limit but are the total error of the experiment and the computational method. The given data are only applicable for comparing calculation methods with each other.

**Figure 16:**
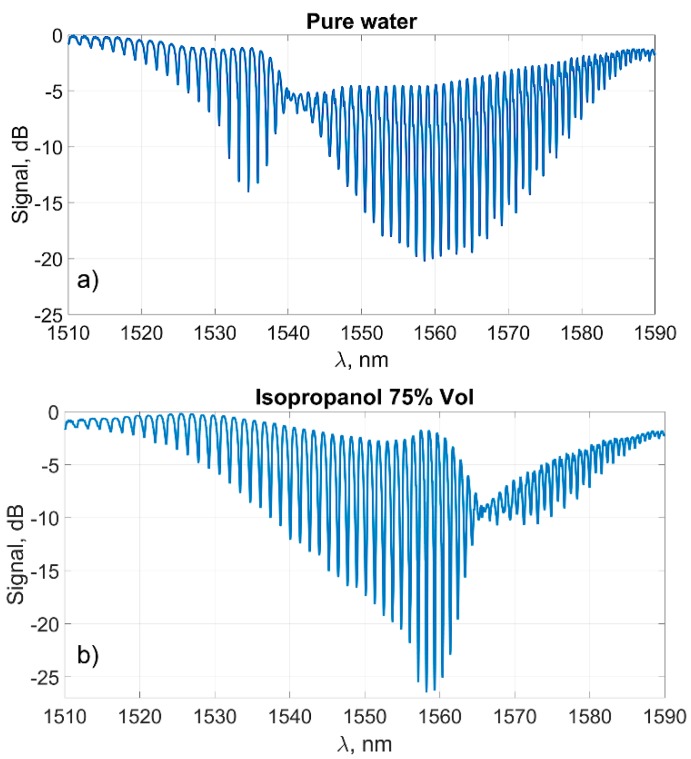
Transmission spectrum of the sensor for two different concentrations of isopropyl alcohol solutions: 0% (**a**), 75% (**b**).

The second experiment was carried out with a similar sensor in order to demonstrate the independence of the processing methods from the parameters of the sensor itself. A wide range of solution concentrations from pure water to a 75% isopropyl alcohol solution was used in the experiment. The complexity of processing in this experiment is due to the fundamentally different shape of the spectral curve around the plasmon resonance wavelength for two very different solution refractive indexes (Figure 16).

Unfortunately, there are no data concerning the changes in the refractive index of isopropyl alcohol solutions depending on the concentration for the near infrared range. On the other hand, the known data for the visible range are quite different [49,50].

**Figure 17:**
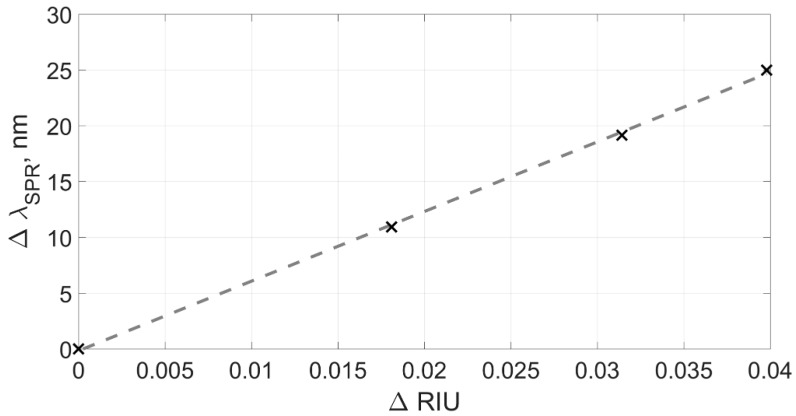
Dependence of the plasmon resonance wavelength shift calculated from the data of the second experiment according to method *f_(2)_*.

We extrapolated the data presented in [49] so it ccould cause the additional errors in the refractive index calculation. Four experimental points with different concentrations were measured in the experiment. The results of experimental data processing using the second method are shown in Figure 17.

Nevertheless, despite the difficulty in processing the fundamentally different spectra (Figure 16), the method demonstrated good results as expected.

The “classical” method using the peak height, as expected, cannot be applied to the analysis of large changes in the refractive index of the environment. More or less predictable changes in its amplitude were observed, while the refractive index changed in the range of not more than 0.007–0.008.

**Figure 18:**
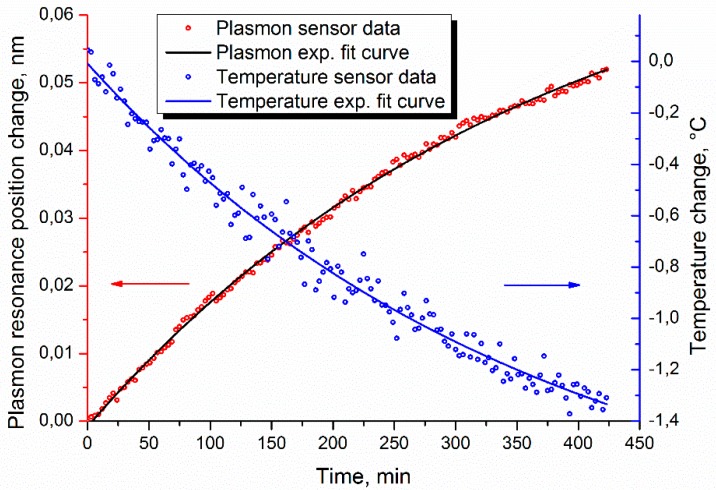
Experimental results for the dynamics of changes in the refractive index during the temperature drift of distilled water.

To reduce the effect of errors associated with the refractive index calculation, preparation and performance of the experiment, we used the data from [26], which were acquired by observing the temperature drift of distilled water. In that experiment, distilled water was heated and then subsequently cooled under natural conditions. The temperature was measured with an independent fiber Bragg grating sensor. In this paper we used data from the second stage of the experiment, where the temperature of the investigated liquid decreased by approximately 1.5 °C, which accordingly led to growth in the liquid’s refractive index. This was observed by the plasmon sensor. Such a cooling process can be approximated with mono-exponential dependence, and the standard deviation of experimental points from the fitting curve can be used to define the sensor resolution. It is known that for distilled water at room temperature, the dispersion of the refractive index is about (−1) × 10^−4^ RIU/°C [51]. As in other experiments mentioned in this paper, a MicronOptics SM-125 Bragg interrogator was used as a spectral interrogation instrument. The experimental data is represented in Figure 18, as well as the corresponding temperature data. The temperature data noise is associated with the intrinsic resolution of the temperature sensor. It is particularly interesting that the best processing results were demonstrated by method 3. This can be explained by the fact that method 3 uses two independent curves, and the calculation results of which are averaged, thus minimizing the error in a small range of changes. The standard deviation of the experimental points from the fitting exponential curve was 5.9 × 10^−4^ nm, which, considering the sensor sensitivity of 576 nm/RIU, establishes the standard deviation in terms of the refractive index equal to 1 × 10^−6^. The triple standard deviation, in fact, corresponds to the detection limit of the sensor [52,53], so the resolution of the sensor can be estimated as 3 × 10^−6^ RIU.

Thus, it can be concluded that to interpret the indications of a plasmon sensor based on a TFBG, it is necessary to use a comprehensive approach to analyzing the spectrum based on taking into account several spectral peaks and the envelope of the total transmission spectrum of the sensor.

## 5. Conclusions

The algorithms for automatic, accurate interpretation of the TFBG-assisted SPR sensor data were developed for the first time. The algorithm based on the comprehensive approach of the sensor’s transmission spectrum analysis permits the determination of the SPR wavelength directly from the spectral constriction position.

The developed methods allow automatic signal processing in a wide dynamic range, taking into account specific transformation in the form of the spectral picture. The efficiency of the developed methods for carrying out measurements in both a narrow and wide dynamic range has been experimentally shown. The method allowed measurements of the refractive index to be conducted with a resolution of 3 × 10^−6^ RIU.

## Figures and Tables

**Table 1:** Comparison of the relative accuracy of different methods for determining changes in the refractive index.

Method	$f_{\left(1\right)}$	$f_{\left(2\right)}$	$f_{\left(3\right)}$	“Classical”
Relative accuracy	$1\times 10^{-4}$	$7.4\times 10^{-5}$	$1\times 10^{-4}$	$2.7\times 10^{-4}$
